# Intercomparison of real-time tailpipe ammonia measurements from vehicles tested over the new world-harmonized light-duty vehicle test cycle (WLTC)

**DOI:** 10.1007/s11356-015-4267-3

**Published:** 2015-03-18

**Authors:** Ricardo Suarez-Bertoa, Alessandro A. Zardini, Velizara Lilova, Daniel Meyer, Shigeru Nakatani, Frank Hibel, Jens Ewers, Michael Clairotte, Leslie Hill, Covadonga Astorga

**Affiliations:** 1 European Commission, Joint Research Centre (JRC), Institute for Energy and Transport (IET), Sustainable Transport Unit, 21027 Ispra, VA Italy; 2 Automotive Test Systems, Emission Engineering, HORIBA Europe GmbH, Hans-Mess-Str. 6, 61440 Oberursel, Germany; 3 CGS Prozessanalytik GmbH, Keltenstraße 3, 85095 Denkendorf, Germany

**Keywords:** Ammonia, Vehicle emissions, FTIR, QCL-IR, BLAQ-Sys, Raw exhaust measurements

## Abstract

Four light-duty vehicles (two diesel, one flex-fuel, and one gasoline vehicle) were tested as part of an intercomparison exercise of the world-harmonized light-duty vehicle test procedure (WLTP) aiming at measuring real-time ammonia emissions from the vehicles’ raw exhaust at the tailpipe. The tests were conducted in the Vehicle Emission Laboratory (VELA) at the European Commission Joint Research Centre (EC-JRC), Ispra, Italy. HORIBA, CGS, and the Sustainable Transport Unit of the Joint Research Centre (JRC) took part in the measurement and analysis of the four vehicles’ exhaust emissions over the world-harmonized light-duty vehicle test cycle class 3, version 5.3 using a HORIBA MEXA 1400 QL-NX, a CGS BLAQ-Sys, and the JRC Fourier transform infrared spectrometer, respectively. The measured ammonia concentrations and the emission profiles revealed that these three instruments are suitable to measure ammonia from the vehicles’ raw exhaust, presenting no significant differences. Furthermore, results showed that measurement of ammonia from the vehicle exhaust using online systems can be performed guaranteeing the reproducibility and repeatability of the results. While no ammonia was detected for any of the two diesel vehicles (even though, one was equipped with a selective catalytic reduction system), we report average ammonia emission factors 8–10 mg/km (average concentrations 20–23 ppm) and 10–12 mg/km (average concentrations 22–24 ppm) for the flex-fuel and gasoline vehicles, respectively.

**Electronic supplementary material:**

The online version of this article (doi:10.1007/s11356-015-4267-3) contains supplementary material, which is available to authorized users.

## Introduction

Ammonia is classified under the European dangerous substances directive (67/548/EEC) as toxic, corrosive, and dangerous for the environment. The US Occupational Safety and Health Administration has set for ammonia an 8-h exposure limit at 25 ppm and a short-term (15 min) exposure level at 35 ppm (Agency for Toxic Substances and Disease Registry A 2004). Its reaction with nitric and sulfuric acid leads to the formation of atmospheric secondary aerosols, namely, ammonium nitrate and ammonium sulfate (Behera and Sharma 2010; Pope et al. 2002). It has been reported that ammonium accounts for up to 17 % of the total PM_2.5_ in the South Coast Air Basin (Kim et al. 2000) and that ammonium, nitrate, and sulfate represent 40 % of the total PM_2.5_ in some European cities (Sillanpää et al. 2006). The deposition of ammonia and/or ammonium salts leads to hypertrophication of waters and acidification of soils with negative effects on nitrogen-containing ecosystems (Bouwman et al. 2002; Erisman et al. 2003; Sutton et al. 2008).

Over the past years, a number of policies have been implemented within Europe that either, directly or indirectly, aims at reducing the emission of ammonia to the atmosphere. These include National Emission Ceilings Directive 2001/81/EC, the Gothenburg Protocol under the United Nations Convention on Long-Range Transboundary Air Pollution (UNECE 1999), and the Intergovernmental Panel on Climate Change Directive (2008/1/EC). Over the last decades, the agriculture and waste management sectors, which are the two main European sources of ammonia, have reduced their emissions by 29 and 24 %, respectively. However, road transportation emissions of ammonia have increased by 378 % in Europe (Technical report No 6/2013), principally due to an increase in the number of vehicles in the fleet and the lack of vehicular ammonia emissions regulations. In order to meet the National Ambient Air Quality Standard for PM_2.5_, the US Environmental Protection Agency (EPA) required regulating ammonia emissions in the case that a State or EPA itself demonstrates that ammonia is a significant contributor to PM_2.5_ formation in an area (Environmental Protection Agency 2011). According to the National Emission Inventory done by the EPA in 2011, ammonia emissions from mobile on-road gasoline light-duty vehicles (LDVs) are the third largest ammonia emission source of the US, after agriculture and fires, and account for 3 % of the total ammonia emissions (EPA 2011 inventory). Previous studies have suggested vehicular emissions to be an important source of ammonia in urban areas (Battye et al. 2003; Chitjian et al. 2000; Ianniello et al. 2010; Kean et al. 2009; Meng et al. 2011; Nowak et al. 2012; Reche et al. 2015, 2012; Whitehead et al. 2007; Yao et al. 2013).

Vehicular emissions of total hydrocarbons (THC), CO, and NOx from LDV are controlled by the legislation ((EC) No 692/2008) (European Commission E 2008). In order to decrease their emissions, the automotive industry introduced the three-way catalyst (TWC). This was a major step towards the vehicular emissions control. Molecular nitrogen is the aimed reaction product during the reduction of NOx over the TWC, but ammonia has been found to be a secondary product during this process. In the TWC, ammonia is formed via steam reforming from hydrocarbons (Whittington et al. 1995) and/or via reaction of nitrogen monoxide (NO) with molecular hydrogen (H_2_) (through reaction 2a or 2b) produced from a water–gas shift reaction between CO and water (Eq. 1) (Barbier Jr and Duprez 1994; Bradow and Stump 1977):

1 $$ \mathrm{C}\mathrm{O} + {\mathrm{H}}_2\mathrm{O}\ \to\ {\mathrm{CO}}_2 + {\mathrm{H}}_2 $$

2a $$ 2\mathrm{NO} + 2\mathrm{C}\mathrm{O} + 3{\mathrm{H}}_2\ \to\ 2{\mathrm{NH}}_3 + 2{\mathrm{CO}}_2 $$

2b $$ 2\mathrm{NO} + 5{\mathrm{H}}_2\ \to\ 2{\mathrm{NH}}_3 + 2{\mathrm{H}}_2\mathrm{O} $$

Ammonia was first identified in vehicle exhaust in 1970s (Shelef and Gandhi 1972), and after that, it is commonly detected in vehicle exhaust, road tunnel air, at roadsides, and in urban air. As a consequence, gasoline LDVs equipped with TWCs are now recognized as an important source of ammonia in the urban areas (Battye et al. 2003; Livingston et al. 2009). Moreover, with the recent introduction of the selective catalytic reduction system (SCR) in heavy-duty vehicles (HDV) and even more recently in diesel LDVs, the potential numbers of vehicles that could emit ammonia may increase. The SCR is an after-treatment system that aims at decreasing NOx emissions by reacting NOx (NO and NO_2_) with NH_3_ on a catalyst surface (reaction 5a–c). Ammonia is formed by the catalytic reaction of urea that is injected into the system. Reactions 3–5c summarized the whole chemical pathway (Gabrielsson 2004):

3 $$ \mathrm{C}\mathrm{O}{\left({\mathrm{NH}}_2\right)}_2\ \to\ {\mathrm{NH}}_3 + \mathrm{HNCO} $$

4 $$ \mathrm{HNCO} + {\mathrm{H}}_2\mathrm{O}\ \to\ {\mathrm{NH}}_3 + {\mathrm{CO}}_2 $$

5a $$ 2{\mathrm{N}\mathrm{H}}_3 + \mathrm{NO} + {\mathrm{N}\mathrm{O}}_2\ \to\ 2\ {\mathrm{N}}_2 + 3{\mathrm{H}}_2\mathrm{O} $$

5b $$ 4{\mathrm{N}\mathrm{H}}_3 + 4\mathrm{NO} + {\mathrm{O}}_2\ \to 4\ {\mathrm{N}}_2 + 6{\mathrm{H}}_2\mathrm{O} $$

5c $$ 8{\mathrm{N}\mathrm{H}}_3 + 6{\mathrm{N}\mathrm{O}}_2\ \to\ 7\ {\mathrm{N}}_2 + 12{\mathrm{H}}_2\mathrm{O} $$

Any possible over-doping of urea and/or catalyst degradation may lead to ammonia emissions. For this reason, the Euro VI emission standards for HDV included a 10 ppm limit for the average emitted tailpipe concentration over the test cycle ((EC) No 582/2011) (European Commission E 2011).

Measuring vehicular ammonia during chassis dynamometer experiments has proven to be an analytical challenge. The standard dynamometer emissions measurement equipment is not adapted to measure this compound (Heeb et al. 2012). Indeed, critical sampling artifacts have been reported when ammonia was analyzed from bag samples. Ammonia can be absorbed in condensed water or react with acidic compounds present in the exhaust or along the system. Previous studies have reported that ammonia sticks, and it is also adsorbed on the surfaces of sampling and analysis equipment, as well as on the dilute exhaust systems’ walls (Durbin et al. 2002; Heeb et al. 2006, 2012, 2008; Mohn et al. 2004). Ammonia is delayed in the constant volume sampler (CVS) making time-resolved ammonia analysis from the CVS tunnel extremely difficult (Mohn et al. 2004). As a result, exhaust tailpipe measurements have been considered to be the most appropriate approach for measuring vehicular ammonia emission on chassis dynamometer tests (Durbin et al. 2002; Heeb et al. 2006, 2012, 2008; Mohn et al. 2004).

It has become necessary to find suitable techniques to measure ammonia emissions from vehicle exhaust during the new world-harmonized light-duty vehicle test procedure (WLTP) that will be soon used for type approval of LDVs in the European Union and potentially other countries who are signatories to the UNECE. Therefore, in the present study, three different analytical techniques, JRC FTIR, HORIBA MEXA 1400 QL-NX, and CGS BLAQ-Sys, were used to evaluate the feasibility of the online ammonia emissions measurement from a series of four different LDVs over the world-harmonized light-duty vehicle test cycle (WLTC).

The Euro VI regulation for HDV specifies Fourier transform infrared spectrometer (FTIR) and laser diode spectrometer) in either in situ or extractive modes as measurement principles to be used for the measurement of ammonia from HD exhaust. Either technique must meet certain criteria, such us a sample path (sampling line, pre-filter(s), and valves) made of stainless steel or polytetrafluoroethylene (PTFE) and heated to 463 ± 10 K (190 ± 10 °C) in order to minimize ammonia losses and sampling artifacts; spectral resolution of the laser (for the near-infrared) or spectral resolution of the ammonia wavelength (for the FTIR) within 0.5 cm^−1^; minimum detection limit of <2 ppm under all conditions of testing; and system response time ≤20 s, among others. The purpose of the campaign was to assess the possibility of the online measurement of ammonia using the proposed analytical methods and also sampling temperatures for the WLTP, both of which are, in some cases, different from what is specified in the Euro VI regulation.

## Experimental

**Fig. 1:**
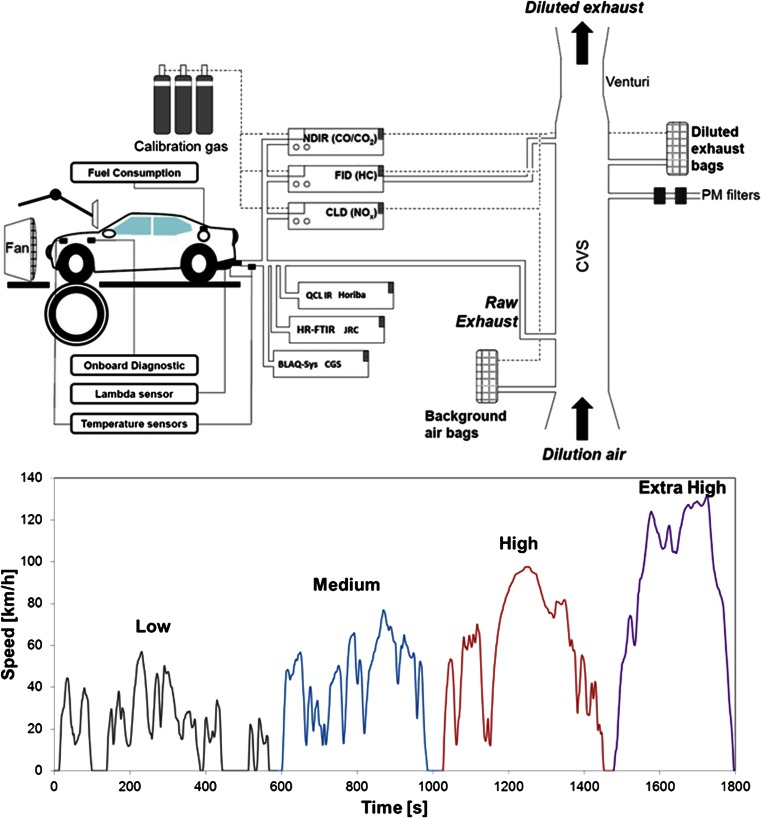
Schematic diagram of the experimental setup (*top*) and world-harmonized light vehicle test cycle (WLTC) (*bottom*). *Each color* represents one of the four phases that form the cycle

Four light-duty vehicles were tested during the online ammonia measurement intercomparison exercise of the WLTP conducted in the Vehicle Emission Laboratory (VELA) at the European Commission Joint Research Centre, Ispra, Italy. Three groups, CGS, HORIBA, and the Sustainable Transport Unit of the JRC took part in the measurement and analysis of the exhaust emissions of the four vehicles over the WLTC class 3, version 5.3 (see Fig. 1) (see section on “World-harmonized light-duty vehicle test cycle”). The test vehicles included two diesel vehicles (DV1 and DV2), one flex-fuel vehicle (FFV), and one gasoline vehicle (GV). The technical description of the vehicles is provided in Table 1. A certified diesel fuel with up to 5 % bio-diesel content (henceforth B5) was used in the two diesel vehicles, DV1 and DV2, while a certified gasoline containing up to 5 % ethanol (hereinafter, E5) was used in the FFV and the GV. The fuel specifications are summarized in Table S1 of the Electronic supplementary material. Vehicles equipped with TWC or selective catalytic reduction (SCR) systems were selected to cover the different potential vehicular ammonia emitters. The DV2 was chosen as a reference diesel vehicle, zero ammonia case, on the basis that no ammonia should be detected (as discussed below), as ammonia is not typically formed in diesel engines nor over diesel particle filter (DPF) systems. Therefore, any ammonia emitted by the diesel vehicle equipped with an SCR (DV1) must result as a slip from this after treatment (i.e., SCR).

**Table 1:** Vehicle description

Denomination	DV1	DV2	FFV	GV
Combustion type	C.I.	C.I.	S.I.	S.I.
Fuel	Diesel (B5)	Diesel (B5)	Gasoline (E5)	Gasoline (E5)
EU emission standard	Euro 6	Euro 5	Euro 5a	Euro 5
After treatment	DPF, SCR	DPF	TWC	TWC
Fuel system	TDI	TD	DI	GDI
Engine displacement (cm^3^)	1968	1560	1596	1390
Odometer (km)	22,362	18,871	24,334	38,541

*S.I*. spark ignition, *C.I.* compression ignition, *DPF* diesel particulate filter, *SCR* selective catalytic reduction, *TWC* three-way catalyst, *TDI* turbocharged direct injection, *TD* turbocharged diesel, *DI* direct injection, *GDI* gasoline direct injection

The VELA 2 facility includes a climatic test cell with controlled temperature and relative humidity (RH) to mimic different ambient conditions (temperature range, −10 to 35 °C; RH, 50 %). Triplicate tests were performed on a chassis dynamometer (inertia range, 454–4,500 kg), designed for two and four-wheel-drive LDVs (two 1.22 m roller benches, Maha GmbH, Germany). The emissions exhaust is fed to a constant volume sampler (CVS, HORIBA, Japan) using a critical Venturi nozzle to regulate the diluted exhaust flow rate (CVS flow range, 3–30 m^3^/min). A series of thermocouples monitored the temperature of the oil, cooling water, exhaust, and ambient conditions. A Universal Exhaust Gas Oxygen-type sensor was connected to the tailpipe to follow the air-to-fuel ratio. The tests were conducted at a test cell temperature of 23 ± 0.1 °C and at 50 ± 2 % RH. The temperature refers not only to the cell temperature but also to the vehicle’s oil temperature, ±1 °C, at the beginning of each test. Vehicles were kept inside the climatic cell under the described conditions for a 20–24 h period, also known as a soak period.

### World-harmonized light-duty vehicle test cycle

In 2009, a road map for a WLTP was proposed by the World Forum for Harmonization of Vehicles Regulations (WP.29) of the United Nations Economic Commission for Europe (UNECE). Since the beginning of the WLTP development process, the European Union had a strong political objective, set by its own legislation (Regulations (EC) 443/2009 and 510/2011) (European Commission E 2009), to implement a new and more realistic test procedure by 2014. This was the major motivation to set the time frame of the whole WLTP and in particular of phase 1.

The formal text for the phase 1a version of the light-duty vehicle Global Technical Regulation (GTR) was adopted by the Working Party on Pollution and Energy transport program in 2013. The GTR specifies globally harmonized performance-related equipment specifications and test procedures. The phase 1a describes the development of the WLTC and the associated test procedure for the common measurement of criteria compounds, CO_2_, fuel, and energy consumption.

Three different driving cycles were developed, on the basis of the vehicle’s power-to-mass ratio and its maximum speed, to represent three different vehicle classes. The vehicles tested in the present study are under class 3 (power/mass >34 kW/ton and maximum speed >120 km/h), which is the highest power and speed class. Figure 1 illustrates version 5.3 of the speed profile for this class.

The WLTC is a cold-start driving cycle, where the vehicle and its components (oil, coolant, catalyst, etc.) are at 23 °C at the beginning of each test. The driving cycle consists of four phases with different speed distributions (see Fig. 1), and it intends to be representative of real-world driving conditions based on real-world vehicle journeys from several countries. The length of the entire cycle is 1,800 s and is comprised of the low-speed (589 s), medium-speed (433 s), high-speed (455 s), and extra-high-speed (323 s) phases. Moreover, it reaches a maximum speed of 131.3 km/h and is about 23.3 km long.

### Analytical instrumentation

#### JRC FTIR spectrometer dedicated to automotive emission

A high-resolution Fourier transform infrared spectrometer (FTIR–MKS Multigas analyzer 2030-HS, Wilmington, MA, USA) allowed measuring the concentrations of up to 20 exhaust compounds by a multivariate calibration based on a factory-developed model. The absorption in the mid-infrared range of several nitrogen species usually emitted in vehicle exhaust is displayed in the Fig. S1 of the Online Resource. As described by this figure, the areas where individual species absorb the infrared (IR) often overlap. For instance, the absorbance of water, displayed with an inversed scale in the upper part of the graphic, can cover the specific absorption wavelength of NO_2_. Consequently, the calibration model has been developed following different steps. Firstly, a principal component analysis (PCA) has been carried out to extract the informative region of the spectra for every individual species. Then, these informative regions were compared in order to isolate the specific wavelength area where no other species absorbs. These specific wavelengths were finally used to compute a multiple least-squares regression (MLR) model which aimed to predict the volume concentration of the compound. For each wavelength selected, the model assumed a linear relationship between concentration and absorption. For each compound, standard gas cylinders of several concentration levels were used to calibrate the model.

Implementation of the FTIR spectrometer for exhaust gas measurement required the acquisition of an averaged background spectrum from N_2_. This daily background spectrum was systematically subtracted from the new spectra registered by the instrument. The calibration of the instrument was based on a factory-developed multivariate model. CO, CO_2_, and NOx measurements from the previously described analyzers were used to check the FTIR calibration model. The limit of detection for ammonia, as well as for the other compounds, was estimated with the values obtained from the measurement of the background air, by assuming three times the standard deviation of the measured value to the averaged value and for ammonia was found to be equal to 0.3 ppm.

FTIR spectrometers dedicated to real-time analysis of automotive emission must have a high acquisition frequency in order to register fast changes of volume concentration resulting from sharp accelerations. The output signal can be used to monitor the fluctuation of pollutant emissions along the speed profile. Furthermore, the CO and/or NO signals were precisely synchronized using the CO or NOx instantaneous measurement at the tailpipe obtained using the non-dispersive infrared (NDIR) or the chemiluminescence detector (CLD), respectively, so that all instruments would be aligned to the very same time.

The JRC FTIR (Multigas Analyzer 2030 HS-MKS, Wilmington, MA) achieved a 5 Hz acquisition frequency with a multipath gas cell of 5.11 m. It is equipped with a Michelson interferometer (spectral resolution, 0.5 cm^−1^; spectral range, 600–3,500 cm^−1^) and a liquid nitrogen cooled mercury cadmium telluride detector. The raw exhaust was sampled directly from the vehicle’s tailpipe with a PTFE-heated line and a pumping system (flow, ca. 10 L min^−1^, T, 191 °C) in order to avoid the wall adsorption and/or dissolution of hydrophilic compounds (i.e., NH_3_, NO_2_, carbonyls, or ethanol) in condensed water. The residence time of the raw exhaust gas in the heated line before the FTIR measurement cell was less than 2 s. The measurement temperature was set to 191 °C, with a working pressure of 1013 (±20) hPa. The compounds were monitored at 5 Hz, averaged, and presented at 1 Hz.

#### HORIBA MEXA 1400 QL-NX. Quantum cascade laser infrared spectrometer

MEXA 1400QL-NX is an analyzer for the direct measurement of four nitrogen compounds (NO, NO_2_, N_2_O, NH_3_) simultaneously in automobile exhaust gas in real time by using IR absorption spectroscopy as the measuring principle. By combining a quantum cascade laser (QCL) light source and a precisely adjusted long dual-path optical cell, it has realized high sensitivity for low concentrations with a limit of detection which complies with the current European legislative requirements. Furthermore, the QCL has a wide dynamic range (i.e., 0–5 ppm to 0–2,000 ppm) for the measurement of ammonia emissions in the exhaust gas. The MEXA 1400 QL-NX has a wavelength resolution close to 0.006 cm^−1^.

The analyzer utilizes a high-resolution spectrum and a high-vacuum optical cell in order to minimize the interference offered by the co-existing gases. Moreover, the ammonia response has been improved by using a vacuum sample transfer line maintained at a temperature of 113 °C, which ensures a shorter residence time and minimum adsorption of ammonia on the surface of the walls. The temperature is optimized to prevent the decomposition of urea or its by-products and ensures a selective measurement of pure ammonia.

The analyzer can be used for the measurement of exhaust gas components from various fuel and engine types. The MEXA 1400QL-NX consists of three main components: a Main Control Unit (MCU), an analyzer unit, and a heated filter (F-01HN). The MCU serves as an instrument for the calculation of emissions and for the display of the measured concentration. The analyzer unit contains the core of the MEXA 1400QL-NX, the sensor including the QCL element, a gas cell and optics, as well as a sample handling system specifically designed for the measurement of ammonia. The heated filter is connected to the analyzer unit via a heated line. The sampling of the exhaust is conducted via a stainless steel sampling probe covered with a heated jacket in order to avoid cold spots. The F-01HN contains a quartz filter element specifically designed to minimize the desorption of the ammonia molecules present in the exhaust gas.

#### CGS-BLAQ-Sys compact test bench

The BLAQ-Sys compact has specifically been designed for measuring traces of ammonia in engine exhaust gases. It contains a heated sampling probe, heated dilution system, and an integrated photo-acoustical analyzer with a QCL.

The technology of the ammonia analyzer is based on the periodic absorption of laser light by ammonia in the sample gas and the subsequent generation of pressure waves. The laser light is generated by a QCL, and the resulting pressure waves are measured with small microphones. The higher the concentration of ammonia, the higher the amplitude of the pressure waves. By calibrating the instrument with a gas mixture containing a known concentration of ammonia, the amplitude of the microphone signal can be associated with a concentration of ammonia in the sample gas.

The design of the measurement cell is optimized for maximum amplification of the signal generated by ammonia and for maximum attenuation of external sound and vibration. A sketch of the measurement concept is shown in Fig. S2 of the Online Resource. The wavelength range that is covered during a measurement was chosen after extensive research on the spectral absorption features of ammonia and the gases that can be expected in the sample gas. For ammonia, the measurement is performed by scanning the laser parameters in such a way that a wavelength range around 965.4 cm^−1^ (10.3 μm) is covered, so the ammonia peak can clearly be recognized and quantified automatically (see Fig. S3 in the Online Resource).

Ammonia molecules tend to stick to the surface of commonly used materials in the exhaust gas measuring technology (e.g., untreated stainless steel). Therefore, only materials exhibiting minimum ammonia adsorption effects like perfluoroalkoxy (PFA), PTFE, and coated stainless steel have been used throughout the complete sampling system and analyzer in the BLAQ-Sys system. This assures that the concentration of ammonia is hardly altered by adsorption/desorption effects before it is measured.

The raw exhaust gas is sampled from the tailpipe with a coated stainless steel CGS extraction probe and transferred via a heated 1/8″ PFA raw gas line (flow, ca 1 L min^−1^; *T*, 190 °C) to the CGS Dilution Unit (DU) (*T*, 100 °C). The dilution module in the DU contains an injector pump, which sucks the raw exhaust gas through a PTFE sample gas filter (PTFE membrane, 1–2 μm; ø, 47 mm). The filter membrane was replaced before every new measuring cycle. Dry instrument air is used to dilute the sample gas flow in the ratio 1:10. The diluted raw exhaust gas is transferred via a heated 3/8″ PFA sample gas line (flow, ca 11 L min^−1^; *T*, 100 °C) to the mobile BLAQ-Sys instrument cabinet. The ammonia analyzer extracts a fraction of the gas flow with its internal pump that is placed behind the heated measurement cell (gas flow, 90 ml min^−1^; *T*, 50 °C; 500 hPa working pressure). The volume of the measurement cell including an inline acoustical filter is 6 ml. The ammonia analyzer has a maximum measurement range of 0–250 ppm, and its detection limit is 0.2 ppm (3 times standard deviation).

During the measurement campaign, the BLAQ-Sys system was calibrated daily over the dilution unit with zero air (synthetic air) and a calibration mixture of 50 ppm ammonia in synthetic air. The time required for performing the calibration measurement was 20 min.

#### Regulated emission measurements

Although the scope of the experiments was to test the feasibility and quality of the vehicular exhaust ammonia measurements, regulated compounds were also measured using standard methodologies defined by the related regulation to assure the performance of the vehicles used. CO, NOx, THC, non-methane hydrocarbons (NMHC), and particulate matter are regulated for vehicle emissions. These pollutants were not analyzed directly at the tailpipe of the vehicle, but after dilution of the raw exhaust. This avoids water condensation in the sampling line and also simulates the dilution process occurring in the atmosphere. For regulatory purposes, there is a collection step from the CVS, either in a Tedlar bag (gaseous pollutant) or on a filter (particulate pollutant). The obtained results are summarized in Table 2.

**Table 2:** Average emission factors (milligrams per kilometer) for the regulated compounds and CO_2_ (grams per kilometer) over the WLTC

Vehicle	THC	THC + NOx	NMHC	CO	NOx	CO_2_	PM
DV1	4 (±3)	188 (±7)	2 (±1)	115 (±9)	184 (±5)	156 (±4)	0.2 (±0.1)
DV2	8 (±3)	467 (±31)	6 (±3)	290 (±19)	458 (±32)	124.5 (±0.7)	0.5 (±0.1)
FFV	80 (±20)	141 (±18)	74 (±20)	470 (±131)	61 (±7)	151.0 (±0.5)	2.0 (±0.1)
GV	29 (±11)	50 (±8)	24 (±10)	309 (±23)	22 (±3)	137.4 (±0.5)	1.4 (±0.2)
Euro 5 S.I.	100	–	68	1,000	60	–	5.0
Euro 6 S.I.	100	–	68	1,000	60	–	4.5
Euro 5 C.I.	–	230	–	500	180	–	5.0
Euro 6 C.I.	–	230	–	500	80	–	4.5

Euro 5 and Euro 6 spark and compression ignition emission limits (units, milligrams per kilometer)

*S.I.* spark ignition emission limits milligrams per kilometer, *C.I.* compression ignition emission limits milligrams per kilometer

The CVS bag sampling method requires the collection of the diluted exhaust with a constant sampling flow rate from the dilution tunnel into a bag, thus, obtaining a representative sample of the total gas which passed through the dilution tunnel during the sampling period. Then, a series of analyzers were connected to the exhaust bags. Once the sampling period was finished, the collected gas was drawn towards the corresponding analyzers. The regulated emissions from all vehicles were measured with the following analyzers: non-dispersive infrared (NDIR for CO/CO_2_), a chemiluminescence (CLD for NOx), and a heated (191 °C) flame ionization detector (FID for THC) (HORIBA, Japan) (see Fig. 1).

Another set of analyzers similar to the one used for gaseous regulated emission measurements was directly connected at the tailpipe of the vehicle. These sensors can monitor online the dynamic volume concentration of the pollutants over the cycle driven with a 1-Hz frequency. This measurement methodology for the raw gas measurement is not indicated in the scope of the legislation; however, the outputs were most informative and useful in the framework of this project. This comprehensive data were correlated with other dynamic parameters, such as the velocity of the vehicle, temperature of the catalyst, and instantaneous fuel consumption. The integration of the recorded signals provided valuable quality control information when compared with the emission factors resulting from the bags, and they proved to be useful for the alignment of the FTIR signals (see FTIR measurements).

The volumetric flow rate of the exhaust (cubic meters per second) was determined by subtracting the variable dilution flow entering the tunnel to the constant total flow inside the tunnel. Mass flows were derived from the exhaust gas flow rates (cubic meter per second) and from the measured concentration (parts per million by volume). Emission factors (milligrams per kilogram) were calculated from the integrated mass flow and the total driving distance of the cycles.

## Results and discussion

### Regulated compounds

The quantification of the regulated compounds emitted by the tested vehicles is of major interest in order to assess the performance and functioning of the tested vehicles. The regulated emissions were measured according to the WLTP GTR. The obtained emission factors together with the Euro 5 and Euro 6 spark and compress ignition emission limits are summarized in Table 2.

**Fig. 2:**
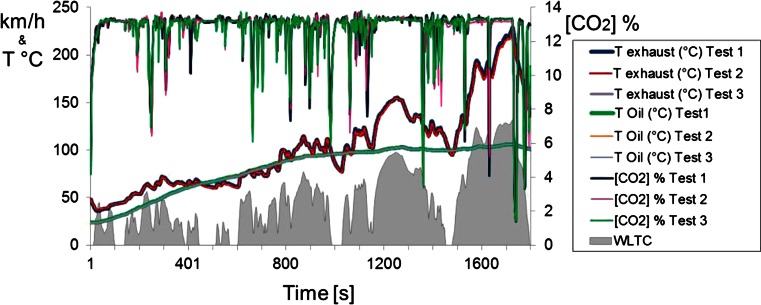
Representation of some setup capabilities (exhaust temperature, CO_2_ emissions, oil temperature) and repeatability for the three tests performed for FFV over the WTLC

Most regulated compounds and ammonia emissions showed very good repeatability, demonstrating the suitability of the testing procedure. An example of the repeatability achieved for CO_2_ emissions, oil temperature, and exhaust temperature can be seen in Fig. 2.

The DV1, a Euro 6 diesel LDV equipped with a DPF and SCR system, emitted on average 184 ± 5 mg/km of NOx. This value doubles the Euro 6 NOx limit (80 mg/km). Therefore, DV1 did not comply with the THC + NOx limit (170 mg/km) either. The high NOx emissions could suggest a malfunctioning of the SCR system (see below). The DV2, a Euro 5 diesel LDV equipped only with a DPF, did not comply with the Euro 5 NOx nor THC + NOx emission limits, with the average emissions being 458 ± 32 and 467 ± 31 mg/km, respectively. In the case of the FFV, average emission factors of NOx and NMHC were within the Euro 5 limits when taking into account the uncertainties (1σ). The other regulated pollutants, THC, CO, and PM, were below emission limits. Finally, the GV complied with the Euro 5 emission standards.

### Ammonia emissions and measurement

**Fig. 3:**
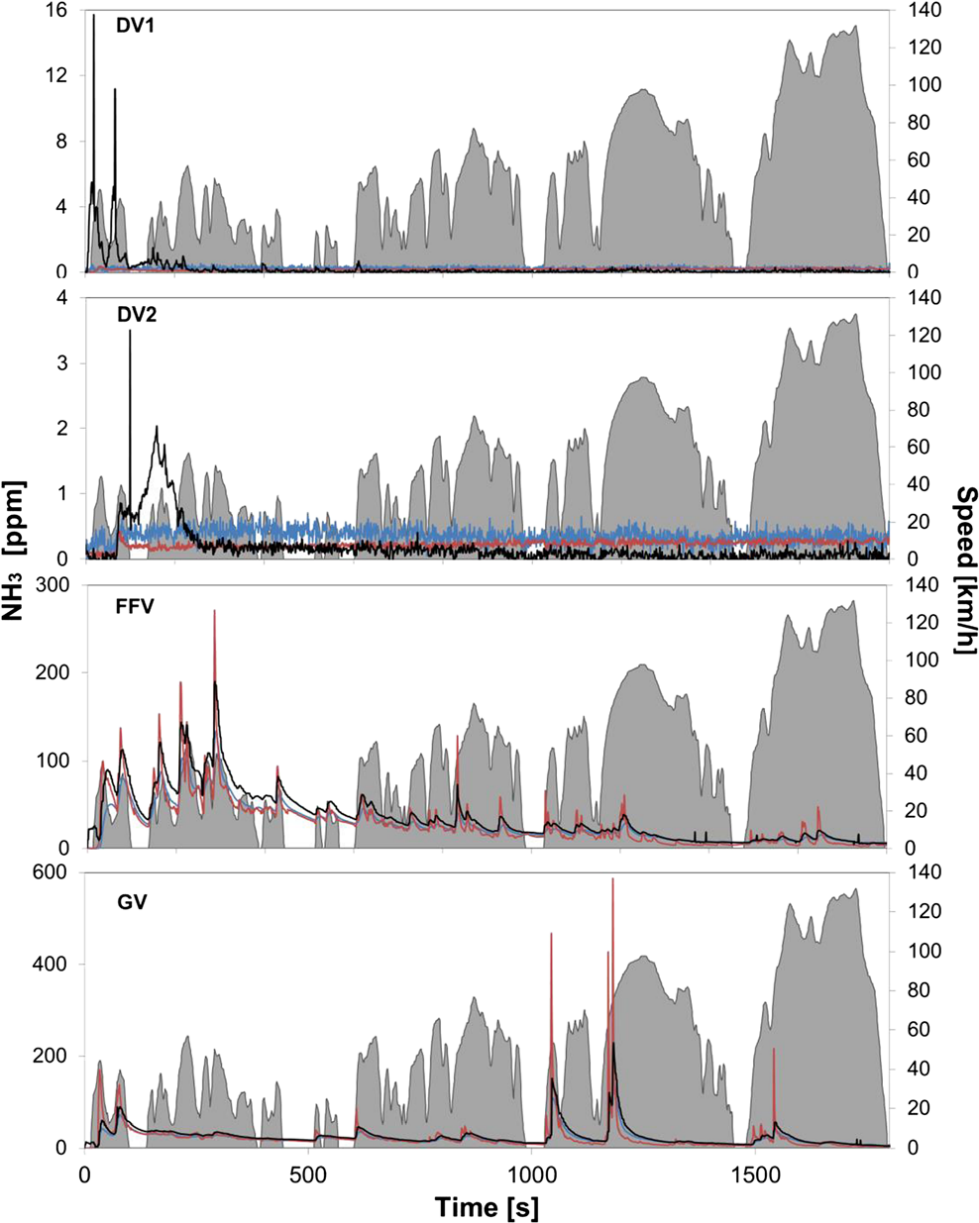
Real-time ammonia emission concentration for vehicles DV1, DV2, FFV, and GV over the WLTC measured by JRC FTIR (*blue*), HORIBA MEXA 1400 QL-NX (*red*), and CGS BLAQ-Sys (*black*)

Figure 3 illustrates the ammonia emission profiles obtained from the four tested vehicles (DV1, DV2, FFV, and GV) using the three analytical instruments described in the section on “Analytical instrumentation”: JRC FTIR, HORIBA MEXA 1400 QL-NX, and CGS BLAQ-Sys. Table 3 shows the average emission factors (mass per distance units) and maximum concentration (parts per million) estimated for each vehicle by each instrument. The presented averages were obtained from the three tests that were performed per vehicle, and the errors refer to one standard deviation.

**Table 3:** Average ammonia emission factors (milligrams per kilogram) and concentration (parts per million) of the three tests performed per vehicle from the four tested vehicles, DV1, DV2, FFV, and GV, using JRC FTIR, HORIBA MEXA 1400 QL-NX and CGS BLAQ-Sys

Vehicle		JRC FTIR	HORIBA QCL-IR	CGS BLAQ-Sys
DV1	mg/km	–	–	0.10 (±0.04)
	Average ppm	0.5 (±0.1)	0.1 (±0.1)	0.2 (±0.1)
	Max ppm	1.0	0.8	6.6
DV2	mg/km	–	–	0.14 (±0.02)
	Average ppm	0.5 (±0.1)	0.1 (±0.1)	0.2 (±0.1)
	Max ppm	1.0	0.5	2.0
FFV	mg/km	8 (±3)	9 (±3)	10 (±5)
	Average ppm	20 (±7)	21 (±7)	23 (±11)
	Max ppm	135	272	190
GV	mg/km	10.3 (±0.5)	11 (±1)	12 (±2)
	Average ppm	22.3 (±0.6)	24 (±1)	24 (±2)
	Max ppm	155.0	587	229

In parentheses are 1σ errors

*Max* maximum concentration registered value (parts per million) during the three tests

**Fig. 4:**
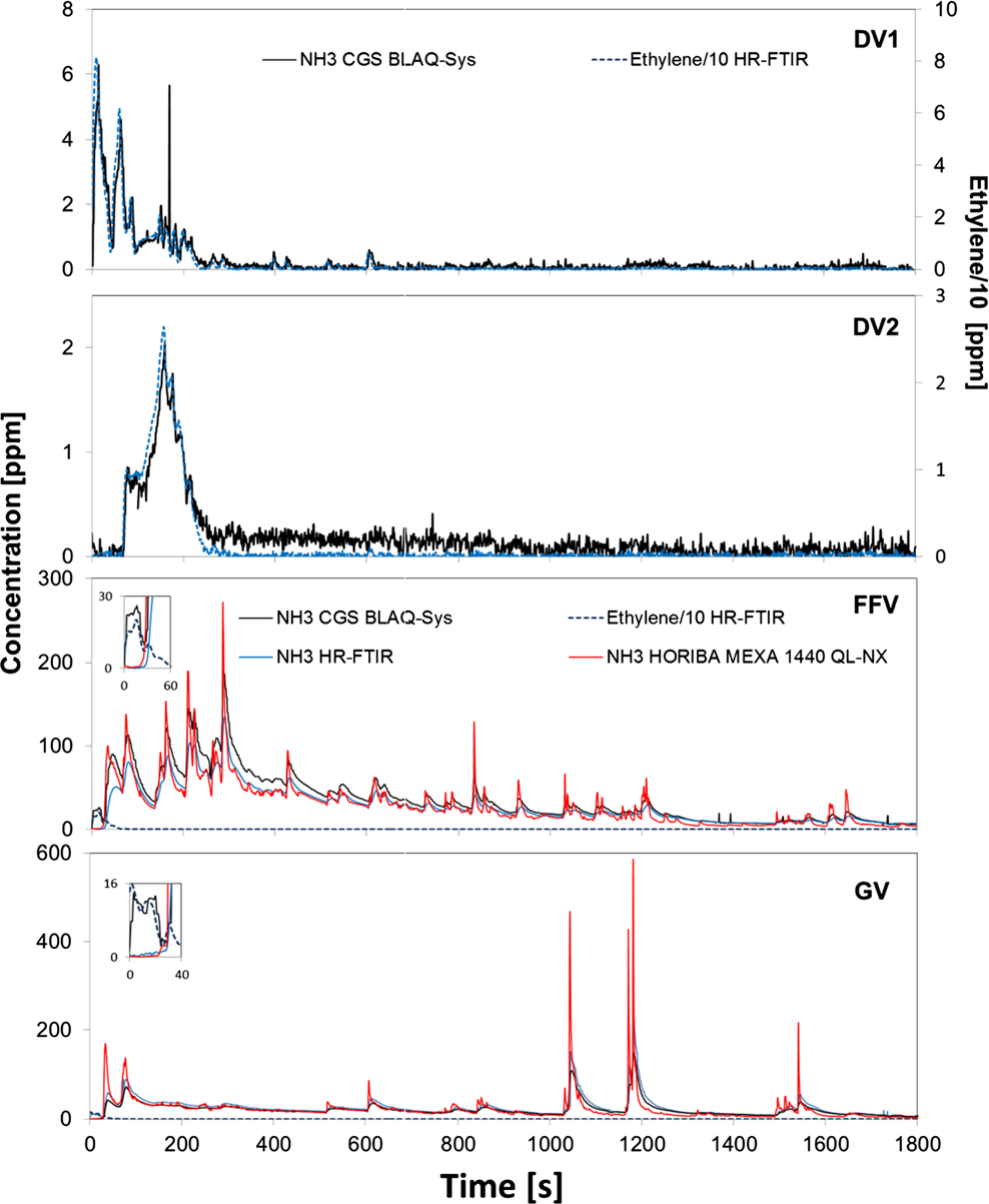
Concentration of ammonia measured by JRC FTIR (*blue*), HORIBA MEXA 1400 QL-NX (*red*), and CGS BLAQ-Sys (*black*), compared with one tenth of the concentration of ethylene, measured by JRC FTIR (*dotted blue*) over the WLTC. Ethylene concentration for DV1 and DV2 is found on the *right axis*

The average ammonia concentrations measured from the raw exhaust of the DV1, a diesel vehicle equipped with a SCR system, by the three instruments were, for the three tests, extremely low (see Table 3). Most of the time, the concentration of ammonia was close or below the limit of detection (LOD) of the JRC FTIR (0.3 ppm) and the HORIBA MEXA 1400 QL-NX (0.2 ppm) instruments. For that reason, emission factors, estimated using these two instruments, are not reported. While the JRC FTIR and the HORIBA MEXA 1400 QL-NX measured concentrations of ammonia close to the LOD over the whole cycle, the CGS BLAQ-Sys detected some ammonia at the very beginning of low phase of the WLTC (see Fig. 3). The comparison of the profile observed by the CGS BLAQ-Sys with those of several other compounds obtained using the JRC FTIR revealed that the CGS BLAQ-Sys had a cross-interference from the ethylene emitted during cold start (see Fig. 4). As Fig. 4 illustrates, this cross-interference was also present during the tests performed with the other vehicles. Still, the average ammonia concentration measured with the BLAQ-Sys did not differ from those obtained with the JRC FTIR and the HORIBA MEXA 1400 QL-NX due to the low concentration and the short emission period of the interfering ethylene.

The high NOx emission factors measured for the DV1 and the absence of any ammonia at its raw exhaust reinforce the hypothesis of a malfunctioning of the vehicle’s SCR system. The DV2 was used as the reference diesel vehicle, from which no ammonia emission was expected to be measured. The JRC FTIR and HORIBA MEXA 1400 QL-NX instruments did not detect any ammonia over the WLTC. The CGS BLAQ-Sys detected low emissions of ammonia. However, the observed profile corresponds exactly to that of the ethylene emitted during the cold start and measured with the JRC FTIR (see Fig. 4), suggesting that the ammonia measured by the CGS BLAQ-Sys was actually the ethylene cross-interference (as for the DV1). Therefore, no ammonia was emitted by DV2 as the other two instruments suggested.

The estimated average ammonia emission factors and as well as the measured concentrations from the FFV and the GV’s raw exhaust were the same, within uncertainties, for the two vehicles and the three instruments (see Table 3). The two vehicles, tested over the WLTC, showed two different ammonia emission profiles that were consistently and evenly reproduced by all three analytical instruments (see Fig. 3). The HORIBA MEXA 1400 QL-NX monitors and reports the ammonia concentrations at a 10-Hz frequency; as a consequence, higher maximum ammonia concentrations were constantly obtained for the two vehicles (272 and 587 ppm for the FFV and the GV, respectively) with this instrument. For the same reason, the peaks that this instrument produces are sharper and better defined. Thus, the average ammonia concentrations obtained with the HORIBA MEXA 1400 QL-NX are the same as those obtained with the JRC FTIR and the CGS BLAQ-Sys. The emission factors obtained from the FFV and the GV over the WLTC at 23 °C ranged from 8 ± 3 ppm to 12 ± 2 mg/km. These results are in line with the ammonia emissions reported by Livingston et al. (2009) (15 ± 13 mg/km) for a fleet of low-emission vehicles (LEV) ultra-low emission vehicles (ULEV) tested over the Federal Test Procedure (FTP) and measured at the tailpipe using an FTIR. The obtained emissions are also in good agreement with those measured by Durbin et al. (2004) for a series of 2000–2001 vehicles tested on the FTP cycle, 9–13 mg/km, using tunable diode laser near-infrared absorption spectrometer. Durbin et al. (2004) observed ammonia emission rates ~5 times higher over the more aggressive US06 cycle compared with the FTP. The obtained average ammonia emission concentrations measured (from 20 ± 7 to 24 ± 2 ppm) and the estimated emission factors are also in very good agreement with the average of the ammonia concentrations and emission factors reported by our group for a series of Euro 5 gasoline vehicles tested over the NEDC at 22 °C (22 ppm and 12 mg/km) (Suarez-Bertoa et al. 2014). In that same study, a similar flex-fuel vehicle was tested using the E5 blend over the NEDC at 22 °C. The reported average concentration of ammonia emitted over the NEDC was 7 ppm (emission factor 4 mg/km), while over the WLTC was around 21 ppm (8–10 mg/km) (see Table 3). Moreover, the maximum ammonia concentration measured for the FFV over the WLTC was, at least, one order of magnitude higher (135–272 ppm) than over the NEDC (14 ppm). These results suggest that the higher concentrations of the ammonia emissions are due to the *dynamicness*, and more realistic nature, of the new cycle.

Our previous work (Suarez-Bertoa et al. 2014) is, to our knowledge, the only study that reports ammonia emission factor and average concentrations for an in-use diesel light-duty vehicle equipped with a SCR system tested on a chassis dynamometer. That vehicle emitted on average 6 ppm of ammonia, with an emission factor of 12 mg/km over the NEDC. Furthermore, Heeb et al. (2011) reported ammonia emission levels ranging from 300 to 1500 mg/h from a diesel engine (3.0 L, 100 kW), operated in the ISO 8178/4 C1. Those results suggest that the introduction of diesel vehicles equipped with SRC will likely result in a larger portion of the fleet that emits ammonia.

## Conclusions

Four light-duty vehicles were tested as part of an intercomparison exercise in the framework of the WLTP. The raw vehicles’ exhaust was analyzed in real time using three different instruments, JRC FTIR, HORIBA MEXA 1400 QL-NX, and CGS BLAQ-Sys. The obtained average ammonia concentrations and the emission profiles reveal that the three instruments are all suitable to measure ammonia from the vehicles’ raw exhaust. The CGS BLAQ-Sys showed a cross-interference from a co-existing compound (ethylene) emitted from the tailpipe during the cold start. This cross-interference has since been solved by the manufacturer. The system quantifies ethylene at a separate wavelength and makes an internal compensation of the ammonia raw signal.

The results showed that all three instruments are in good agreement, showing no significant differences. The three instruments also show a very good reproducibility. It is then perfectly feasible to measure ammonia in the vehicle exhaust with an online method, guaranteeing the reproducibility and repeatability of the results.

The HORIBA MEXA 1400 QL-NX was operating at 113 °C as opposed to 190 °C of the other instruments. The results indicate that temperature of the sampling line and analyzer is not important as long as there is no condensation.

Little can be said about the diesel vehicle 1 (DV1) ammonia emissions, since a malfunctioning of the SCR system is suspected. The FFV and the GV showed similar average ammonia emission factors (8–12 mg/km) compared with those reported for gasoline LDVs in previous studies (Durbin et al. 2004; Livingston et al. 2009; Suarez-Bertoa et al. 2014). Following the introduction of the different emission legislations, emissions of NOx and CO have substantially decreased over the past years. However, the ammonia emissions, which are not regulated, have remained comparable to those that have been reported for spark ignition light-duty vehicles during the last decade. Since ammonia has been shown to be present in the emissions from spark ignition vehicles and compression ignition vehicles equipped with SRC systems, and the available techniques to measure ammonia from the vehicles have proved to be robust, the introduction of an ammonia emission regulation for light-duty spark and compression ignition vehicles should be thoughtfully considered. Furthermore, implementation and adjustment of complementary devices, such as an oxidizing catalyst right after the SCR (Koebel et al. 2000) or after the TWC, or an appropriate feedback and control technology of the air/fuel ratio that also looks out for ammonia emissions (Kitagawa et al. 2000) may be necessary when LDV ammonia emission limits are introduced.

## Electronic supplementary material

Below is the link to the electronic supplementary material.

ESM 1 (DOCX 301 kb)
