# Supplementary material for: Intercomparison of real-time tailpipe ammonia measurements from vehicles tested over the new world-harmonized light-duty vehicle test cycle (WLTC)
Source: Environ Sci Pollut Res Int. 2015 Mar 18;22(10):7450–60. doi: 10.1007/s11356-015-4267-3 (PMC4432089; doi:10.1007/s11356-015-4267-3)

Intercomparison of real-time tailpipe ammonia measurements from vehicles tested over the new Worldwide harmonized Light-duty vehicle Test Cycle (WLTC)

Environmental Science and Pollution Research

Ricardo Suarez-Bertoa^1*^, Alessandro A. Zardini^1^, Velizara Lilova^2^, Daniel Meyer^3^, Shigeru Nakatani^2^, Frank Hibel^3^, Jens Ewers^3^, Michael Clairotte^1^, Leslie Hill^2^ and Covadonga Astorga^1*^.

*^1^European Commission Joint Research Centre Ispra, Institute for Energy and Transport, Sustainable Transport Unit, 21027 Ispra (VA), Italy.*

*^2^HORIBA Europe GmbH, Emission Engineering, Automotive Test Systems, Hans-Mess-Str. 6, 61440 Oberursel, Germany.*

*^3^CGS Prozessanalytik GmbH, Keltenstraße 3, D-85095 Denkendorf, Germany*.

*Corresponding authors:

E-mail addresses: [ricardo.suarez-bertoa@jrc.ec.europa.eu](mailto:ricardo.suarez-bertoa@jrc.ec.europa.eu) (R. Suarez-Bertoa),

[covadonga.astorga-llorens@jrc.ec.europa.eu](mailto:covadonga.astorga-llorens@jrc.ec.europa.eu%20) (C. Astorga)

**Supplementary Material**

**Table S1.** Fuels specifications.

|  | **Gasoline** |  |  |  | **Diesel** |  |  |
| --- | --- | --- | --- | --- | --- | --- | --- |
| **Parameter** | **Method** | **Unit** | **E5** | **Parameter** | **Method** | **Unit** | **B5** |
| RON | EN ISO 5164 | - | 101.2 | Cetane Number | EN ISO 5165 | - | 52.4 |
| MON | EN ISO 5163 | - | 90.4 | Density at 15 °C | EN ISO 3675 | - | 835.5 |
| Density at 15 °C | EN ISO 3675 | kg/m^3^ | 751.8 | Sulfur | EN ISO 20846 | kg/m^3^ | 3.9 |
| DVPE | EN 13016-1 | kPa | 65.0 | Viscosity at 40 °C | EN ISO 3104 | mm^2^/s | 2.53 |
| Sulfur | EN ISO 20846 | mg/kg | <3.0 | Lubricity at 60 °C | ISO/DIS 12156 | µm | 127 |
| Net Heating Value | ASTM D3338 | MJ/kg | 42.020 | Net Heating Value | ASTM D4868 | MJ/kg | 42.94 |
| Carbon | ASTM D3343 | % (V) | 85.14 | Carbon | ASTM D5291 | % (V) | 86.1 |
| Hydrogen | ASTM D3343 | % (V) | 13.16 | Hydrogen | ASTM D5622 | % (V) | 13.1 |
| Oxygen | EN 13132 | % (V) | 1.7 | Oxygen | Calculated | % (V) | 0.7 |
| Ethanol | EN 13132 | % (V) | 4.7 |  |  |  |  |

**Fig. S1.** FTIR spectra of typical vehicular emitted nitrogen species and H_2_O.


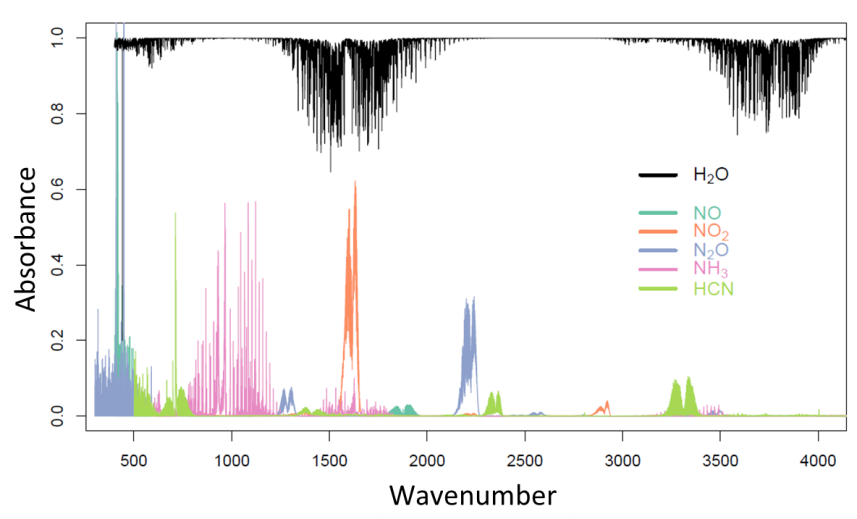


**Fig. S2.** Sketch of the measurement concept the BLAQ-Sys Compact. The modulated laser light is directed from the left to the right through the measurement cell, resulting in a pressure modulation that is measured by microphones; the pressure amplitude is proportional to the NH_3_ concentration.


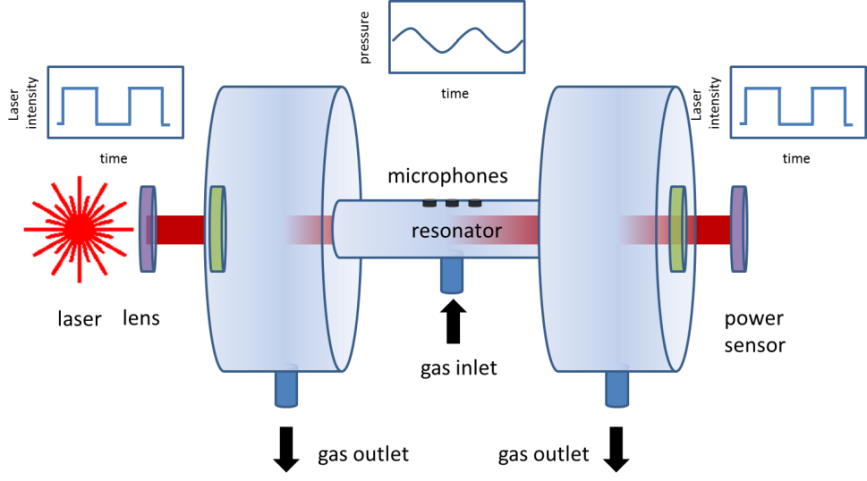


**Fig. S3.** CGS- BLAQ-Sys. Absorption spectra from NH_3_, H_2_O and CO_2_, the vertical axis represents the fraction of the light that is absorbed in 1 cm of ambient air at a pressure of 0.5 bar.
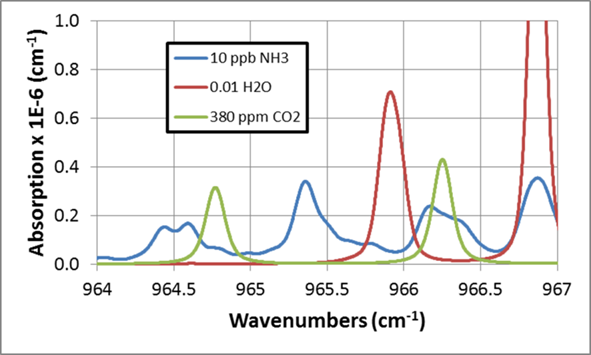

Supplement: Supplementary file 1 — (DOCX 301 kb) [file 11356_2015_4267_MOESM1_ESM.docx]
